# Supplementary material for: MED-EL hearing solution registry: An examination of the strengths and limitations of a cochlear implant registry
Source: PLoS One. 2025 Oct 27;20(10):e0335345. doi: 10.1371/journal.pone.0335345 (PMC12558452; doi:10.1371/journal.pone.0335345)
Supplement: S1-3 Tables — (DOCX) [file pone.0335345.s001.docx]

# **MED-EL Hearing Solution Registry: An examination of the strengths and limitations of a cochlear implant registry**

Uwe Baumann, Tobias Weissgerber, Andreas Radeloff, Karin A. Koinig, Magdalena Breu, Jasmine Rinnofner, Vera Lohnherr, Stefan Dazert, Christiane Völter, Ilona Anderson, Joachim Müller, Daniel Polterauer

## **Raw data used in the manuscript**

[Table 1: Wearing Time (daily CI use in hours per day) 2](#_Toc202170013)

[Table 2: FMS data (Monosyllables % correct) 5](#_Toc202170014)

[Table 3: FNum data (Numerals % correct) 8](#_Toc202170015)

Table 1: Wearing Time (daily CI use in hours per day)

| **ID** | **side** | **age** | **implant** | **array** | **audio-processor** | **period** | **wearing time/day** |
| --- | --- | --- | --- | --- | --- | --- | --- |
| 5 | R | 68 |  | FLEXSOFT | SONNET 2 EAS | 3-months | More than 12 h |
| 6 | R | 53 | SYNCHRONY 2 | FLEX28 | SONNET 2 | 6-months | More than 12 h |
| 26 | L | 33 | SYNCHRONY 2 | STANDARD | SONNET 2 | 1-year | More than 12 h |
| 28 | L | 55 | SYNCHRONY 2 | FLEXSOFT | SONNET 2 | 1-year | More than 12 h |
| 35 | R | 52 | SYNCHRONY 2 | FLEXSOFT | SONNET 2 EAS | 3-months | 3 h to 5 h 59 min |
| 56 | L | 2 | SYNCHRONY 2 | FLEX28 | SONNET 2 | 6-months | More than 12 h |
| 80 | R | 30 | SYNCHRONY 2 | FLEX28 | SONNET 2 | 1-year | More than 12 h |
| 83 | L | 9 | SYNCHRONY 2 | FLEXSOFT | SONNET 2 | 1-year | 3 h to 5 h 59 min |
| 102 | R | 81 | SYNCHRONY 2 PIN | FLEX28 | SONNET 2 EAS | 3-months | More than 12 h |
| 109 | R | 74 |  | STANDARD | SONNET 2 | 3-months | More than 12 h |
| 131 | R | 73 | SYNCHRONY 2 | STANDARD | SONNET 2 | pre-OP | 6 h to 8 h 59 min |
| 145 | L | 4 |  |  | SONNET 2 | 6-months | 6 h to 8 h 59 min |
| 199 | L | 29 | SYNCHRONY 2 | FLEX28 | SONNET 2 | 3-months | More than 12 h |
| 205 | R | 56 |  | STANDARD | SONNET 2 | 3-months | 3 h to 5 h 59 min |
| 216 | L | 62 | SYNCHRONY 2 | FLEX24 | SONNET 2 | 1-year | More than 12 h |
| 219 | R | 74 | SYNCHRONY 2 PIN | FLEXSOFT | SONNET 2 | 6-months | 3 h to 5 h 59 min |
| 233 | R | 40 |  | FLEXSOFT | SONNET 2 | 3-months | More than 12 h |
| 236 | R | 66 | SYNCHRONY PIN | STANDARD | SONNET 2 | 1-year | More than 12 h |
| 239 | R | 58 | SYNCHRONY 2 | FLEX28 | SONNET 2 | 1-year | 9 h to 11 h 59 min |
| 251 | R | 49 | SYNCHRONY 2 | FLEXSOFT | SONNET 2 | 6-months | More than 12 h |
| 268 | L | 22 | SYNCHRONY 2 | FLEXSOFT | SONNET 2 | 6-months | 9 h to 11 h 59 min |
| 283 | L | 61 | SYNCHRONY 2 | FLEX28 | SONNET 2 | 1-year | More than 12 h |
| 319 | R | 77 | SYNCHRONY PIN | FLEX28 | SONNET 2 | 1-year | 6 h to 8 h 59 min |
| 322 | R | 63 | SYNCHRONY 2 | FLEXSOFT | SONNET 2 | 6-months | More than 12 h |
| 322 | L | 66 | SYNCHRONY 2 | FLEXSOFT | SONNET 2 | pre-OP | More than 12 h |
| 325 | R | 40 | SYNCHRONY 2 | STANDARD | SONNET 2 | 3-months | 9 h to 11 h 59 min |
| 335 | R | 22 | SYNCHRONY 2 | FLEXSOFT | SONNET 2 | 6-months | More than 12 h |
| 335 | L | 23 |  | FLEXSOFT | SONNET 2 | pre-OP | More than 12 h |
| 336 | R | 55 |  | STANDARD | SONNET 2 | 3-months | More than 12 h |
| 359 | R | 71 | SYNCHRONY 2 PIN | FLEX28 | SONNET 2 | 1-year | More than 12 h |
| 381 | R | 80 | SYNCHRONY 2 | FLEXSOFT | SONNET 2 | 6-months | 9 h to 11 h 59 min |
| 398 | R | 79 | SYNCHRONY 2 | FLEX26 | SONNET 2 | 1-year | More than 12 h |
| 412 | R | 1 | SYNCHRONY 2 | FLEX28 | SONNET 2 | 1-year | More than 12 h |
| 420 | R | 73 | SYNCHRONY 2 | FLEX28 | SONNET 2 | 3-months | More than 12 h |
| 427 | R | 78 | SYNCHRONY 2 PIN | FLEXSOFT | SONNET 2 | 1-year | More than 12 h |
| 460 | L | 55 | SYNCHRONY 2 PIN | FLEXSOFT | SONNET 2 | 6-months | 3 h to 5 h 59 min |
| 472 | R | 67 | SYNCHRONY 2 | FLEX26 | SONNET 2 | 6-months | More than 12 h |
| 475 | L | 14 | SYNCHRONY 2 | FLEXSOFT | SONNET 2 | 1-year | More than 12 h |
| 487 | R | 70 | SYNCHRONY PIN | FLEXSOFT | SONNET 2 | 6-months | More than 12 h |
| 501 | R | 47 | SYNCHRONY 2 PIN | FLEX28 | SONNET 2 | 6-months | More than 12 h |
| 518 | R | 56 | SYNCHRONY 2 | FLEXSOFT | SONNET 2 | 1-year | More than 12 h |
| 525 | L | 60 | SYNCHRONY 2 PIN | FLEX28 | SONNET 2 | 1-year | 9 h to 11 h 59 min |
| 530 | L | 58 | SYNCHRONY 2 | FLEXSOFT | SONNET 2 | 6-months | 9 h to 11 h 59 min |
| 556 | R | 55 | SYNCHRONY 2 | FLEX26 | SONNET 2 | 6-months | More than 12 h |
| 562 | R | 9 | SYNCHRONY 2 | FLEXSOFT | SONNET 2 | 6-months | More than 12 h |
| 567 | L | 1 | SYNCHRONY 2 | STANDARD | SONNET 2 | 6-months | More than 12 h |
| 567 | R | 1 |  | STANDARD | SONNET 2 | 3-months | More than 12 h |
| 576 | L | 57 | SYNCHRONY 2 | FLEX28 | SONNET 2 | 6-months | 3 h to 5 h 59 min |
| 577 | R | 0 |  | STANDARD | SONNET 2 | 6-months | 3 h to 5 h 59 min |
| 577 | L | 0 |  | STANDARD | SONNET 2 | 3-months | 3 h to 5 h 59 min |
| 581 | L | 5 | SYNCHRONY 2 | FLEX28 | SONNET 2 | 6-months | More than 12 h |
| 615 | L | 66 | SYNCHRONY | STANDARD | SONNET 2 | 6-months | More than 12 h |
| 626 | R | 77 |  | STANDARD | SONNET 2 | 3-months | More than 12 h |
| 635 | R | 5 | SYNCHRONY | FLEXSOFT | SONNET 2 | 1-year | 9 h to 11 h 59 min |
| 638 | L | 17 | SYNCHRONY 2 | FLEXSOFT | SONNET 2 | 6-months | 0 h to 2 h 59 min |
| 666 | L | 75 | SYNCHRONY 2 | FLEXSOFT | SONNET 2 | 6-months | 6 h to 8 h 59 min |
| 675 | R | 43 |  | FLEXSOFT | SONNET 2 | 3-months | 9 h to 11 h 59 min |
| 694 | L | 0 | SYNCHRONY 2 | FLEX28 | SONNET 2 | 6-months | 9 h to 11 h 59 min |
| 701 | L | 46 | SYNCHRONY 2 | FLEX28 | SONNET 2 EAS | 1-year | 3 h to 5 h 59 min |
| 713 | L | 73 | SYNCHRONY 2 PIN | FLEX28 | SONNET 2 | 6-months | 0 h to 2 h 59 min |
| 724 | L | 72 | SYNCHRONY 2 | STANDARD | SONNET 2 | pre-OP | 6 h to 8 h 59 min |
| 728 | L | 5 |  | FLEX28 | SONNET 2 | 6-months | More than 12 h |
| 728 | R | 5 |  | FLEX28 | SONNET 2 | pre-OP | More than 12 h |
| 741 | L | 56 |  | FLEX28 | SONNET 2 | 3-months | More than 12 h |
| 741 | R | 57 |  | MEDIUM | SONNET 2 | pre-OP | More than 12 h |
| 753 | L | 73 |  |  | SONNET 2 | 1-year | 6 h to 8 h 59 min |
| 755 | L | 31 | SYNCHRONY 2 | FLEXSOFT | SONNET 2 | 3-months | 6 h to 8 h 59 min |
| 758 | R | 36 |  | STANDARD | SONNET 2 | 3-months | More than 12 h |
| 762 | L | 57 | SYNCHRONY 2 | FLEX24 | SONNET 2 EAS | 1-year | More than 12 h |
| 767 | R | 67 |  | FLEX28 | SONNET 2 EAS | 6-months | 3 h to 5 h 59 min |
| 768 | R | 66 | SYNCHRONY 2 | FLEX28 | SONNET 2 EAS | 1-year | More than 12 h |
| 772 | L | 76 | SYNCHRONY 2 | FLEX28 | SONNET 2 | 6-months | 9 h to 11 h 59 min |
| 772 | R | 79 | SYNCHRONY 2 | FLEX28 | SONNET 2 | 3-months | 9 h to 11 h 59 min |
| 806 | R | 53 | SYNCHRONY 2 | FLEXSOFT | SONNET 2 | 6-months | More than 12 h |
| 807 |  | 65 | SYNCHRONY 2 | FLEXSOFT | SONNET 2 | 1-year | More than 12 h |
| 816 | L | 46 |  | FLEXSOFT | SONNET 2 EAS | 6-months | More than 12 h |
| 837 | R | 12 |  | FLEXSOFT | SONNET 2 EAS | 6-months | 9 h to 11 h 59 min |
| 841 | R | 72 | SYNCHRONY 2 | FLEXSOFT | SONNET 2 | 1-year | 6 h to 8 h 59 min |
| 855 | L | 11 |  | FLEX28 | SONNET 2 | 6-months | 9 h to 11 h 59 min |
| 861 | L | 59 | SYNCHRONY 2 | FLEX26 | SONNET 2 | 3-months | 9 h to 11 h 59 min |
| 870 | L | 57 | SYNCHRONY 2 PIN | STANDARD | SONNET 2 | 1-year | More than 12 h |
| 875 | R | 59 | SYNCHRONY 2 | STANDARD | SONNET 2 | pre-OP | More than 12 h |
| 879 | L | 33 | SYNCHRONY 2 | FLEXSOFT | SONNET 2 | 3-months | More than 12 h |
| 887 | R | 58 | SYNCHRONY 2 | STANDARD | SONNET 2 | 6-months | More than 12 h |
| 888 | L | 54 |  | FLEXSOFT | SONNET 2 | 6-months | More than 12 h |
| 889 | L | 55 | SYNCHRONY 2 | FLEX26 | SONNET 2 | 1-year | 9 h to 11 h 59 min |
| 893 | L | 48 | SYNCHRONY 2 | STANDARD | SONNET 2 | 6-months | 6 h to 8 h 59 min |
| 907 | L | 6 | SYNCHRONY 2 PIN | STANDARD | SONNET 2 | 3-months | 9 h to 11 h 59 min |
| 908 | L | 35 |  | FLEX28 | SONNET 2 | 3-months | More than 12 h |
| 915 | L | 41 | SYNCHRONY 2 | STANDARD | SONNET 2 | 3-months | 3 h to 5 h 59 min |
| 929 | L | 70 | SYNCHRONY 2 | FLEX24 | SONNET 2 | 3-months | 3 h to 5 h 59 min |
| 954 | R | 25 | SYNCHRONY 2 | FLEX28 | SONNET 2 | 1-year | More than 12 h |
| 959 | R | 63 | SYNCHRONY 2 | FLEXSOFT | SONNET 2 | pre-OP | More than 12 h |
| 962 | R | 20 | SYNCHRONY 2 | FLEX28 | SONNET 2 | 3-months | More than 12 h |
| 970 | L | 57 |  | STANDARD | SONNET 2 | 3-months | 6 h to 8 h 59 min |
| 985 | R | 71 | SYNCHRONY 2 | FLEX26 | SONNET 2 | 1-year | More than 12 h |
| 1004 | L | 67 | SYNCHRONY 2 | FLEXSOFT | SONNET 2 | 1-year | More than 12 h |
| 1010 | L | 66 | SYNCHRONY 2 | FLEX28 | SONNET 2 | 1-year | More than 12 h |
| 1013 | L | 54 | SYNCHRONY 2 | STANDARD | SONNET 2 | 1-year | More than 12 h |
| 1016 | R | 15 | SYNCHRONY 2 | STANDARD | SONNET 2 | 3-months | More than 12 h |
| 1023 | R | 73 | SYNCHRONY 2 | FLEX28 | SONNET 2 | 1-year | More than 12 h |
| 1043 | L | 62 | SYNCHRONY 2 | FLEX28 | SONNET 2 | 1-year | More than 12 h |
| 1054 | L | 14 | SYNCHRONY 2 | FLEX28 | SONNET 2 | 1-year | More than 12 h |
| 1079 | R | 65 | SYNCHRONY | FLEXSOFT | SONNET 2 | 1-year | More than 12 h |
| 1097 | R | 62 | SYNCHRONY 2 | FLEX26 | SONNET 2 | 1-year | More than 12 h |
| 1115 | R | 64 | SYNCHRONY 2 | FLEX28 | SONNET 2 | 1-year | More than 12 h |
| 1117 | R | 86 | SYNCHRONY 2 | FLEX28 | SONNET 2 | 1-year | More than 12 h |
| 1135 | R | 59 | SYNCHRONY 2 | FLEXSOFT | SONNET 2 | 6-months | More than 12 h |
| 1169 | L | 70 |  | STANDARD | SONNET 2 | 3-months | 3 h to 5 h 59 min |
| 1201 | L | 30 | SYNCHRONY 2 PIN | FLEXSOFT | SONNET 2 | 1-year | More than 12 h |
| 1202 | R | 51 | SYNCHRONY 2 | STANDARD | SONNET 2 | 3-months | More than 12 h |
| 1203 | R | 67 |  | FLEX28 | SONNET 2 EAS | 3-months | More than 12 h |
| 1213 | L | 59 | SYNCHRONY PIN | STANDARD | SONNET 2 EAS | 1-year | More than 12 h |
| 1246 | R | 56 |  | STANDARD | SONNET 2 | 6-months | More than 12 h |
| 1252 | R | 30 | SYNCHRONY | FLEX28 | SONNET 2 | 1-year | More than 12 h |
| 1252 | L | 31 | SYNCHRONY 2 | FLEX28 | SONNET 2 | 6-months | More than 12 h |
| 1272 | L | 22 | SYNCHRONY 2 | STANDARD | SONNET 2 | 1-year | 9 h to 11 h 59 min |
| 1284 | L | 2 | SYNCHRONY 2 | FLEX28 | SONNET 2 | 6-months | 3 h to 5 h 59 min |
| 1288 | L | 83 | SYNCHRONY 2 | FLEX28 | SONNET 2 | 6-months | 0 h to 2 h 59 min |
| 1294 | L | 71 | SYNCHRONY 2 | STANDARD | SONNET 2 | 1-year | More than 12 h |
| 1303 | L | 35 | SYNCHRONY 2 | FLEX28 | SONNET 2 | 1-year | 9 h to 11 h 59 min |
| 1307 | R | 85 | SYNCHRONY 2 | FLEX28 | SONNET 2 EAS | 6-months | More than 12 h |
| 1310 | R | 38 |  | FLEX28 | SONNET 2 | 6-months | 9 h to 11 h 59 min |
| 1318 | L | 50 | SYNCHRONY 2 | FLEXSOFT | SONNET 2 EAS | 6-months | More than 12 h |
| 1338 | L | 52 |  | STANDARD | SONNET 2 EAS | 6-months | 6 h to 8 h 59 min |
| 1344 | R | 30 | SYNCHRONY 2 | STANDARD | SONNET 2 | 6-months | More than 12 h |
| 1353 | L | 51 |  | FLEX28 | SONNET 2 | 3-months | 9 h to 11 h 59 min |
| 1377 | R | 4 | SYNCHRONY 2 | FLEXSOFT | SONNET 2 | 1-year | More than 12 h |
| 1379 | R | 1 | SYNCHRONY 2 | FLEXSOFT | SONNET 2 | 3-months | 9 h to 11 h 59 min |
| 1380 | R | 77 | SYNCHRONY 2 | STANDARD | SONNET 2 | 6-months | 9 h to 11 h 59 min |
| 1394 | R | 6 | SYNCHRONY 2 | STANDARD | SONNET 2 | 6-months | 3 h to 5 h 59 min |
| 1406 | R | 1 | SYNCHRONY 2 | STANDARD | SONNET 2 | 1-year | 9 h to 11 h 59 min |
| 1422 | L | 36 | SYNCHRONY 2 | STANDARD | SONNET 2 | 3-months | More than 12 h |
| 1438 | L | 29 | SYNCHRONY 2 | FLEX28 | SONNET 2 | 3-months | More than 12 h |
| 1448 | L | 86 | CONCERTO | STANDARD | SONNET 2 | 6-months | More than 12 h |
| 1460 | R | 60 |  | FLEXSOFT | SONNET 2 | 3-months | More than 12 h |
| 1485 | R | 59 | SYNCHRONY 2 | FLEX28 | SONNET 2 | 6-months | More than 12 h |
| 1488 | L | 37 | SYNCHRONY 2 | FLEX28 | SONNET 2 | 6-months | More than 12 h |
| 1491 | R | 61 |  | FLEX28 | SONNET 2 | 3-months | 3 h to 5 h 59 min |
| 1506 | R | 86 | SYNCHRONY 2 | FLEX28 | SONNET 2 | pre-OP | More than 12 h |
| 1507 | R | 86 | SYNCHRONY PIN | STANDARD | SONNET 2 | 6-months | 9 h to 11 h 59 min |
| 1509 | R | 54 | SYNCHRONY | FLEX28 | SONNET 2 | 1-year | More than 12 h |
| 1519 | R | 1 | SYNCHRONY 2 | FLEXSOFT | SONNET 2 | 6-months | 9 h to 11 h 59 min |
| 1530 | R | 9 |  | FLEXSOFT | SONNET 2 | 3-months | 9 h to 11 h 59 min |
| 1539 | R | 1 | SYNCHRONY 2 | STANDARD | SONNET 2 | 3-months | 9 h to 11 h 59 min |
| 1547 | L | 75 | SYNCHRONY 2 PIN | FLEXSOFT | SONNET 2 | 1-year | 0 h to 2 h 59 min |
| 1551 | R | 81 |  | FLEXSOFT | SONNET 2 | 3-months | 9 h to 11 h 59 min |
| 1570 | L | 58 | SYNCHRONY 2 | FLEXSOFT | SONNET 2 | 3-months | 9 h to 11 h 59 min |
| 1588 | L | 4 | SYNCHRONY 2 | STANDARD | SONNET 2 | 1-year | 9 h to 11 h 59 min |
| 1589 | R | 59 | SYNCHRONY 2 | FLEXSOFT | SONNET 2 | 3-months | 3 h to 5 h 59 min |
| 1592 | L | 77 | SYNCHRONY | STANDARD | SONNET 2 | 1-year | More than 12 h |
| 1601 | R | 53 |  | FLEX28 | SONNET 2 | 6-months | More than 12 h |
| 1603 | L | 55 | SYNCHRONY PIN | STANDARD | SONNET 2 | 3-months | More than 12 h |
| 1612 | R | 37 | SYNCHRONY 2 | FLEX24 | SONNET 2 | 6-months | More than 12 h |
| 1622 | R | 78 | SYNCHRONY 2 | FLEX28 | SONNET 2 | 1-year | 9 h to 11 h 59 min |
| 1623 | L | 51 | SYNCHRONY 2 | FLEX28 | SONNET 2 | 6-months | More than 12 h |
| 1628 | R | 15 |  | FLEXSOFT | SONNET 2 | 3-months | 9 h to 11 h 59 min |
| 1657 | L | 52 | SYNCHRONY 2 | FLEX24 | SONNET 2 | 1-year | 9 h to 11 h 59 min |
| 1669 | L | 63 | SYNCHRONY 2 | FLEX28 | SONNET 2 | 6-months | 0 h to 2 h 59 min |

Table 2: FMS data (Monosyllables % correct)

| **ID** | **side** | **age** | **implant** | **array** | **audio-processor** | **period** | **FMS** |
| --- | --- | --- | --- | --- | --- | --- | --- |
| 6 | R | 53 | SYNCHRONY 2 | FLEX28 | SONNET 2 | 3-months | 85% |
| 6 | R | 53 | SYNCHRONY 2 | FLEX28 | SONNET 2 | 6-months | 95% |
| 10 | L | 77 | SYNCHRONY | FLEX28 |  | pre-OP | 0% |
| 45 | L | 12 |  | FLEXSOFT | SONNET 2 EAS | 3-months | 25% |
| 80 | L | 29 | SYNCHRONY 2 | FLEX28 |  | pre-OP | 5% |
| 80 | L | 29 | SYNCHRONY 2 | FLEX28 | SONNET 2 | 6-months | 80% |
| 80 | L | 29 | SYNCHRONY 2 | FLEX28 | SONNET 2 | 1-year | 85% |
| 80 | R | 30 | SYNCHRONY 2 | FLEX28 | SONNET 2 EAS | 3-months | 70% |
| 84 | R | 67 |  | FLEXSOFT | SONNET 2 | 3-months | 20% |
| 138 | L | 31 | SYNCHRONY | FLEX24 | SONNET | pre-OP | 45% |
| 144 | R | 18 |  | FLEXSOFT | SONNET 2 EAS | 3-months | 0% |
| 174 | R | 66 |  | STANDARD | SONNET 2 EAS | 3-months | 5% |
| 199 | L | 29 | SYNCHRONY 2 | FLEX28 | SONNET 2 | 3-months | 80% |
| 199 | L | 29 | SYNCHRONY 2 | FLEX28 | SONNET 2 | 6-months | 95% |
| 199 | L | 29 | SYNCHRONY 2 | FLEX28 | SONNET 2 | 1-year | 70% |
| 216 | L | 62 | SYNCHRONY 2 | FLEX24 |  | pre-OP | 5% |
| 216 | L | 62 | SYNCHRONY 2 | FLEX24 | SONNET 2 EAS | 3-months | 20% |
| 216 | L | 62 | SYNCHRONY 2 | FLEX24 | SONNET 2 | 6-months | 60% |
| 216 | L | 62 | SYNCHRONY 2 | FLEX24 | SONNET 2 | 1-year | 65% |
| 236 | R | 66 | SYNCHRONY PIN | STANDARD | SONNET 2 | 1-year | 35% |
| 247 | R | 61 |  | STANDARD | SONNET 2 | 3-months | 10% |
| 283 | L | 61 | SYNCHRONY 2 | FLEX28 | SONNET 2 | pre-OP | 0% |
| 283 | L | 61 | SYNCHRONY 2 | FLEX28 | SONNET 2 | 6-months | 35% |
| 283 | L | 61 | SYNCHRONY 2 | FLEX28 | SONNET 2 | 1-year | 40% |
| 294 | L | 43 | CONCERTO | FLEXSOFT |  | pre-OP | 0% |
| 310 | R | 39 | SYNCHRONY | FLEX28 |  | pre-OP | 50% |
| 322 | R | 63 | SYNCHRONY 2 | FLEXSOFT | SONNET 2 | 6-months | 55% |
| 322 | R | 63 | SYNCHRONY 2 | FLEXSOFT | SONNET 2 | 1-year | 20% |
| 324 | L | 33 | PULSAR | FLEXSOFT | SONNET | pre-OP | 15% |
| 348 | R | 58 | SYNCHRONY | FLEX28 | SONNET | pre-OP | 20% |
| 362 | L | 75 | SYNCHRONY | FLEX28 | SONNET | pre-OP | 0% |
| 381 | R | 80 | SYNCHRONY 2 | FLEXSOFT | SONNET 2 | 6-months | 39% |
| 398 | L | 77 | SYNCHRONY 2 | FLEX28 | SONNET 2 | 1-year | 70% |
| 398 | R | 79 | SYNCHRONY 2 | FLEX26 |  | pre-OP | 45% |
| 398 | R | 79 | SYNCHRONY 2 | FLEX26 | SONNET 2 | 3-months | 80% |
| 398 | R | 79 | SYNCHRONY 2 | FLEX26 | SONNET 2 | 6-months | 85% |
| 427 | R | 78 | SYNCHRONY 2 PIN | FLEXSOFT | SONNET 2 | pre-OP | 35% |
| 433 | L | 58 | SYNCHRONY | FLEX28 | SONNET | pre-OP | 15% |
| 438 | R | 83 | SYNCHRONY 2 | FLEX28 |  | pre-OP | 0% |
| 438 | R | 83 | SYNCHRONY 2 | FLEX28 | SONNET 2 | 6-months | 50% |
| 438 | R | 83 | SYNCHRONY 2 | FLEX28 | SONNET 2 | 1-year | 65% |
| 447 | L | 75 | PULSAR | FLEX24 |  | pre-OP | 20% |
| 472 | R | 67 | SYNCHRONY 2 | FLEX26 |  | pre-OP | 5% |
| 472 | R | 67 | SYNCHRONY 2 | FLEX26 | SONNET 2 | 3-months | 85% |
| 472 | R | 67 | SYNCHRONY 2 | FLEX26 | SONNET 2 | 6-months | 95% |
| 472 | R | 67 | SYNCHRONY 2 | FLEX26 | SONNET 2 | 1-year | 78% |
| 505 | R | 56 | SYNCHRONY | FLEX28 | SONNET | pre-OP | 35% |
| 510 | R | 59 |  | FLEX28 | SONNET 2 | 3-months | 20% |
| 511 | L | 64 | SYNCHRONY | FLEX28 |  | pre-OP | 0% |
| 518 | R | 56 | SYNCHRONY 2 | FLEXSOFT | SONNET 2 | 6-months | 65% |
| 518 | R | 56 | SYNCHRONY 2 | FLEXSOFT | SONNET 2 | 1-year | 70% |
| 556 | R | 55 | SYNCHRONY 2 | FLEX26 | SONNET 2 | 3-months | 60% |
| 556 | R | 55 | SYNCHRONY 2 | FLEX26 | SONNET 2 | 6-months | 60% |
| 576 | L | 57 | SYNCHRONY 2 | FLEX28 | SONNET 2 | 3-months | 55% |
| 576 | L | 57 | SYNCHRONY 2 | FLEX28 | SONNET 2 | 6-months | 65% |
| 576 | L | 57 | SYNCHRONY 2 | FLEX28 | SONNET 2 | 1-year | 65% |
| 620 | R | 47 | CONCERTO | FLEX24 | SONNET | pre-OP | 35% |
| 626 | R | 77 |  | STANDARD | SONNET 2 | 1-year | 20% |
| 645 | L | 35 | SYNCHRONY | FLEX28 |  | pre-OP | 20% |
| 675 | R | 43 |  | FLEXSOFT | SONNET 2 | 1-year | 40% |
| 677 | L | 79 |  | FLEX28 | SONNET 2 | 6-months | 50% |
| 707 | R | 47 | SONATA | FLEX24 | SONNET | pre-OP | 20% |
| 762 | L | 57 | SYNCHRONY 2 | FLEX24 |  | pre-OP | 35% |
| 762 | L | 57 | SYNCHRONY 2 | FLEX24 | SONNET 2 EAS | 3-months | 40% |
| 762 | L | 57 | SYNCHRONY 2 | FLEX24 | SONNET 2 EAS | 6-months | 50% |
| 762 | L | 57 | SYNCHRONY 2 | FLEX24 | SONNET 2 EAS | 1-year | 80% |
| 768 | R | 66 | SYNCHRONY 2 | FLEX28 | SONNET 2 | 3-months | 65% |
| 768 | R | 66 | SYNCHRONY 2 | FLEX28 | SONNET 2 EAS | 6-months | 65% |
| 768 | R | 66 | SYNCHRONY 2 | FLEX28 | SONNET 2 EAS | 1-year | 75% |
| 772 | L | 76 | SYNCHRONY 2 | FLEX28 | SONNET 2 | 6-months | 45% |
| 772 | L | 76 | SYNCHRONY 2 | FLEX28 | SONNET 2 | 1-year | 60% |
| 772 | R | 79 | SYNCHRONY 2 | FLEX28 | SONNET 2 | 3-months | 70% |
| 793 | L | 44 | SYNCHRONY | FLEX28 |  | pre-OP | 28% |
| 813 | R | 55 | SYNCHRONY | FLEX24 |  | pre-OP | 10% |
| 815 | R | 72 |  | STANDARD | SONNET 2 | 3-months | 35% |
| 861 | R | 59 | SYNCHRONY | FLEX26 |  | pre-OP | 5% |
| 861 | L | 59 | SYNCHRONY 2 | FLEX26 | SONNET 2 | 3-months | 75% |
| 874 | R | 78 | SYNCHRONY | FLEX28 | SONNET | pre-OP | 10% |
| 884 | L | 39 | SYNCHRONY | FLEX28 |  | pre-OP | 0% |
| 889 | L | 55 | SYNCHRONY 2 | FLEX26 | SONNET 2 | 3-months | 50% |
| 889 | L | 55 | SYNCHRONY 2 | FLEX26 | SONNET 2 | 1-year | 75% |
| 929 | L | 70 | SYNCHRONY 2 | FLEX24 | SONNET 2 | 6-months | 60% |
| 929 | L | 70 | SYNCHRONY 2 | FLEX24 | SONNET 2 | 1-year | 80% |
| 937 | R | 42 | CONCERTO | FLEX28 | SONNET 2 | pre-OP | 0% |
| 937 | R | 42 | CONCERTO | FLEX28 | SONNET 2 | 3-months | 0% |
| 948 | L | 63 |  | FLEXSOFT | SONNET 2 | pre-OP | 15% |
| 954 | R | 25 | SYNCHRONY 2 | FLEX28 | SONNET 2 | 3-months | 80% |
| 954 | R | 25 | SYNCHRONY 2 | FLEX28 | SONNET 2 | 6-months | 80% |
| 954 | R | 25 | SYNCHRONY 2 | FLEX28 | SONNET 2 | 1-year | 95% |
| 973 | L | 80 | SYNCHRONY | FLEX28 |  | pre-OP | 0% |
| 980 | L | 88 | SYNCHRONY | FLEX28 |  | pre-OP | 10% |
| 985 | R | 71 | SYNCHRONY 2 | FLEX26 |  | pre-OP | 10% |
| 985 | R | 71 | SYNCHRONY 2 | FLEX26 | SONNET 2 | 3-months | 90% |
| 985 | R | 71 | SYNCHRONY 2 | FLEX26 | SONNET 2 | 6-months | 85% |
| 985 | R | 71 | SYNCHRONY 2 | FLEX26 | SONNET 2 | 1-year | 90% |
| 987 | R | 36 | SYNCHRONY | FLEX28 | SONNET | pre-OP | 0% |
| 1010 | L | 66 | SYNCHRONY 2 | FLEX28 | SONNET 2 | 3-months | 15% |
| 1010 | L | 66 | SYNCHRONY 2 | FLEX28 | SONNET 2 | 6-months | 45% |
| 1010 | L | 66 | SYNCHRONY 2 | FLEX28 | SONNET 2 | 1-year | 65% |
| 1016 | R | 15 | SYNCHRONY 2 | STANDARD | SONNET 2 | 6-months | 60% |
| 1023 | R | 73 | SYNCHRONY 2 | FLEX28 | SONNET 2 | 3-months | 50% |
| 1023 | R | 73 | SYNCHRONY 2 | FLEX28 | SONNET 2 | 6-months | 60% |
| 1043 | L | 62 | SYNCHRONY 2 | FLEX28 | SONNET 2 | 6-months | 70% |
| 1043 | L | 62 | SYNCHRONY 2 | FLEX28 | SONNET 2 | 1-year | 75% |
| 1054 | L | 14 | SYNCHRONY 2 | FLEX28 | SONNET 2 | 6-months | 65% |
| 1054 | L | 14 | SYNCHRONY 2 | FLEX28 | SONNET 2 | 1-year | 35% |
| 1085 | L | 71 | SYNCHRONY | FLEX28 | SONNET | pre-OP | 0% |
| 1094 | L | 64 | SYNCHRONY | FLEX28 |  | pre-OP | 10% |
| 1097 | R | 62 | SYNCHRONY 2 | FLEX26 |  | pre-OP | 0% |
| 1097 | R | 62 | SYNCHRONY 2 | FLEX26 | SONNET 2 | 3-months | 60% |
| 1097 | R | 62 | SYNCHRONY 2 | FLEX26 | SONNET 2 | 6-months | 95% |
| 1097 | R | 62 | SYNCHRONY 2 | FLEX26 | SONNET 2 | 1-year | 90% |
| 1115 | R | 64 | SYNCHRONY 2 | FLEX28 | SONNET 2 | 3-months | 20% |
| 1115 | R | 64 | SYNCHRONY 2 | FLEX28 | SONNET 2 | 6-months | 70% |
| 1115 | R | 64 | SYNCHRONY 2 | FLEX28 | SONNET 2 | 1-year | 55% |
| 1117 | R | 86 | SYNCHRONY 2 | FLEX28 | SONNET 2 EAS | 3-months | 25% |
| 1117 | R | 86 | SYNCHRONY 2 | FLEX28 | SONNET 2 | 1-year | 60% |
| 1135 | R | 59 | SYNCHRONY 2 | FLEXSOFT | SONNET 2 | 6-months | 50% |
| 1162 | R | 56 |  | FLEXSOFT | SONNET 2 | 3-months | 40% |
| 1172 | R | 61 |  | FLEXSOFT | SONNET 2 | 3-months | 15% |
| 1173 | L | 80 | SYNCHRONY 2 | FLEX28 | SONNET 2 | 3-months | 85% |
| 1173 | L | 80 | SYNCHRONY 2 | FLEX28 | SONNET 2 | 6-months | 75% |
| 1181 | R | 42 | SYNCHRONY | FLEX24 |  | pre-OP | 10% |
| 1202 | R | 51 | SYNCHRONY 2 | STANDARD | SONNET 2 | 6-months | 50% |
| 1252 | R | 30 | SYNCHRONY | FLEX28 |  | pre-OP | 0% |
| 1252 | L | 31 | SYNCHRONY 2 | FLEX28 | SONNET 2 | 3-months | 61% |
| 1301 | R | 64 |  | FLEX28 | SONNET 2 EAS | 3-months | 95% |
| 1307 | R | 85 | SYNCHRONY 2 | FLEX28 | SONNET 2 EAS | 3-months | 80% |
| 1307 | R | 85 | SYNCHRONY 2 | FLEX28 | SONNET 2 EAS | 6-months | 65% |
| 1329 | L | 80 |  | FLEXSOFT | SONNET 2 EAS | pre-OP | 0% |
| 1329 | L | 80 |  | FLEXSOFT | SONNET 2 EAS | 3-months | 15% |
| 1344 | R | 30 | SYNCHRONY 2 | STANDARD | SONNET 2 | 3-months | 15% |
| 1344 | R | 30 | SYNCHRONY 2 | STANDARD | SONNET 2 | 6-months | 75% |
| 1399 | R | 44 |  | FLEXSOFT | SONNET 2 | 3-months | 10% |
| 1401 | R | 53 | SYNCHRONY | FLEX28 |  | pre-OP | 50% |
| 1425 | L | 79 | SYNCHRONY 2 | STANDARD | SONNET 2 | 3-months | 0% |
| 1436 | R | 59 | CONCERTO | FLEX24 | SONNET | pre-OP | 30% |
| 1438 | L | 29 | SYNCHRONY 2 | FLEX28 | SONNET 2 | 3-months | 30% |
| 1454 | R | 88 | SYNCHRONY | FLEX28 | SONNET | pre-OP | 8% |
| 1485 | R | 59 | SYNCHRONY 2 | FLEX28 |  | pre-OP | 30% |
| 1485 | R | 59 | SYNCHRONY 2 | FLEX28 | SONNET 2 | 3-months | 80% |
| 1485 | R | 59 | SYNCHRONY 2 | FLEX28 | SONNET 2 | 6-months | 90% |
| 1488 | L | 37 | SYNCHRONY 2 | FLEX28 | SONNET 2 | 3-months | 45% |
| 1488 | L | 37 | SYNCHRONY 2 | FLEX28 | SONNET 2 | 1-year | 55% |
| 1570 | L | 58 | SYNCHRONY 2 | FLEXSOFT |  | pre-OP | 5% |
| 1570 | L | 58 | SYNCHRONY 2 | FLEXSOFT | SONNET 2 | 3-months | 55% |
| 1622 | R | 78 | SYNCHRONY 2 | FLEX28 | SONNET 2 | 6-months | 70% |
| 1622 | R | 78 | SYNCHRONY 2 | FLEX28 | SONNET 2 | 1-year | 50% |
| 1623 | L | 51 | SYNCHRONY 2 | FLEX28 | SONNET 2 | 6-months | 80% |
| 1657 | L | 52 | SYNCHRONY 2 | FLEX24 |  | pre-OP | 40% |
| 1657 | L | 52 | SYNCHRONY 2 | FLEX24 | SONNET 2 | 3-months | 45% |
| 1657 | L | 52 | SYNCHRONY 2 | FLEX24 | SONNET 2 | 1-year | 60% |
| 1669 | L | 63 | SYNCHRONY 2 | FLEX28 | SONNET 2 | 3-months | 15% |
| 1669 | L | 63 | SYNCHRONY 2 | FLEX28 | SONNET 2 | 6-months | 30% |
| 1669 | L | 63 | SYNCHRONY 2 | FLEX28 | SONNET 2 | 1-year | 35% |
| 1680 | L | 76 | SYNCHRONY | FLEX24 |  | pre-OP | 30% |

Table 3: FNum data (Numerals % correct)

| **ID** | **side** | **age** | **implant** | **array** | **audio-processor** | **period** | **FNum** |
| --- | --- | --- | --- | --- | --- | --- | --- |
| 6 | L | 49 | SYNCHRONY | FLEX28 |  | pre-OP | 0% |
| 6 | R | 53 | SYNCHRONY 2 | FLEX28 | SONNET 2 | 3-months | 100% |
| 10 | L | 77 | SYNCHRONY | FLEX28 |  | pre-OP | 40% |
| 45 | L | 12 |  | FLEXSOFT | SONNET 2 EAS | 3-months | 100% |
| 80 | L | 29 | SYNCHRONY 2 | FLEX28 |  | pre-OP | 78% |
| 80 | L | 29 | SYNCHRONY 2 | FLEX28 | SONNET 2 | 6-months | 100% |
| 80 | R | 30 | SYNCHRONY 2 | FLEX28 | SONNET 2 EAS | 3-months | 100% |
| 84 | R | 67 |  | FLEXSOFT | SONNET 2 | 3-months | 100% |
| 138 | L | 31 | SYNCHRONY | FLEX24 | SONNET | pre-OP | 100% |
| 144 | R | 18 |  | FLEXSOFT | SONNET 2 EAS | 3-months | 70% |
| 174 | R | 66 |  | STANDARD | SONNET 2 EAS | 3-months | 50% |
| 199 | L | 29 | SYNCHRONY 2 | FLEX28 | SONNET 2 | pre-OP | 70% |
| 199 | L | 29 | SYNCHRONY 2 | FLEX28 | SONNET 2 | 3-months | 90% |
| 216 | L | 62 | SYNCHRONY 2 | FLEX24 | SONNET 2 EAS | 3-months | 90% |
| 216 | L | 62 | SYNCHRONY 2 | FLEX24 | SONNET 2 | 6-months | 90% |
| 216 | L | 62 | SYNCHRONY 2 | FLEX24 | SONNET 2 | 1-year | 100% |
| 229 | L | 57 |  | STANDARD | SONNET 2 | 3-months | 0% |
| 247 | R | 61 |  | STANDARD | SONNET 2 | 3-months | 80% |
| 283 | L | 61 | SYNCHRONY 2 | FLEX28 | SONNET 2 | 3-months | 70% |
| 283 | L | 61 | SYNCHRONY 2 | FLEX28 | SONNET 2 | 6-months | 100% |
| 294 | L | 43 | CONCERTO | FLEXSOFT |  | pre-OP | 0% |
| 310 | R | 39 | SYNCHRONY | FLEX28 |  | pre-OP | 100% |
| 322 | R | 63 | SYNCHRONY 2 | FLEXSOFT | SONNET 2 | 3-months | 90% |
| 322 | R | 63 | SYNCHRONY 2 | FLEXSOFT | SONNET 2 | 6-months | 100% |
| 322 | R | 63 | SYNCHRONY 2 | FLEXSOFT | SONNET 2 | 1-year | 100% |
| 324 | L | 33 | PULSAR | FLEXSOFT | SONNET | pre-OP | 100% |
| 344 | L | 55 | SYNCHRONY | FLEX28 |  | pre-OP | 0% |
| 348 | R | 54 | CONCERTO | FLEX24 | SONNET | pre-OP | 70% |
| 362 | L | 75 | SYNCHRONY | FLEX28 | SONNET | pre-OP | 0% |
| 381 | R | 80 | SYNCHRONY 2 | FLEXSOFT |  | pre-OP | 20% |
| 381 | R | 80 | SYNCHRONY 2 | FLEXSOFT | SONNET 2 | 3-months | 40% |
| 381 | R | 80 | SYNCHRONY 2 | FLEXSOFT | SONNET 2 | 6-months | 90% |
| 398 | L | 77 | SYNCHRONY 2 | FLEX28 | SONNET 2 | 1-year | 100% |
| 398 | R | 79 | SYNCHRONY 2 | FLEX26 |  | pre-OP | 60% |
| 398 | R | 79 | SYNCHRONY 2 | FLEX26 | SONNET 2 | 3-months | 100% |
| 398 | R | 79 | SYNCHRONY 2 | FLEX26 | SONNET 2 | 6-months | 100% |
| 433 | L | 58 | SYNCHRONY | FLEX28 | SONNET | pre-OP | 70% |
| 438 | R | 83 | SYNCHRONY 2 | FLEX28 |  | pre-OP | 78% |
| 438 | R | 83 | SYNCHRONY 2 | FLEX28 | SONNET 2 | 6-months | 100% |
| 447 | L | 75 | PULSAR | FLEX24 |  | pre-OP | 90% |
| 472 | R | 67 | SYNCHRONY 2 | FLEX26 | SONNET 2 | 3-months | 100% |
| 472 | R | 67 | SYNCHRONY 2 | FLEX26 | SONNET 2 | 6-months | 100% |
| 505 | R | 56 | SYNCHRONY | FLEX28 | SONNET | pre-OP | 100% |
| 511 | L | 64 | SYNCHRONY | FLEX28 |  | pre-OP | 17% |
| 518 | R | 56 | SYNCHRONY 2 | FLEXSOFT | SONNET 2 | 3-months | 50% |
| 518 | R | 56 | SYNCHRONY 2 | FLEXSOFT | SONNET 2 | 6-months | 90% |
| 556 | R | 55 | SYNCHRONY 2 | FLEX26 | SONNET 2 | 3-months | 100% |
| 556 | R | 55 | SYNCHRONY 2 | FLEX26 | SONNET 2 | 6-months | 100% |
| 576 | L | 57 | SYNCHRONY 2 | FLEX28 | SONNET 2 | 3-months | 100% |
| 576 | L | 57 | SYNCHRONY 2 | FLEX28 | SONNET 2 | 6-months | 100% |
| 620 | R | 47 | CONCERTO | FLEX24 | SONNET | pre-OP | 90% |
| 626 | R | 77 |  | STANDARD | SONNET 2 | 1-year | 100% |
| 645 | L | 35 | SYNCHRONY | FLEX28 |  | pre-OP | 100% |
| 670 | L | 38 | SONATA | FLEXSOFT | SONNET | pre-OP | 0% |
| 675 | R | 43 |  | FLEXSOFT | SONNET 2 | 1-year | 100% |
| 677 | L | 79 |  | FLEX28 | SONNET 2 | 6-months | 80% |
| 707 | R | 47 | SONATA | FLEX24 | SONNET | pre-OP | 80% |
| 755 | L | 31 | SYNCHRONY 2 | FLEXSOFT | SONNET 2 | 3-months | 30% |
| 762 | L | 57 | SYNCHRONY 2 | FLEX24 | SONNET 2 EAS | 3-months | 100% |
| 762 | L | 57 | SYNCHRONY 2 | FLEX24 | SONNET 2 EAS | 6-months | 100% |
| 762 | L | 57 | SYNCHRONY 2 | FLEX24 | SONNET 2 EAS | 1-year | 90% |
| 768 | R | 66 | SYNCHRONY 2 | FLEX28 | SONNET 2 | 3-months | 70% |
| 768 | R | 66 | SYNCHRONY 2 | FLEX28 | SONNET 2 EAS | 6-months | 90% |
| 768 | R | 66 | SYNCHRONY 2 | FLEX28 | SONNET 2 EAS | 1-year | 100% |
| 772 | L | 76 | SYNCHRONY 2 | FLEX28 | SONNET 2 | 6-months | 100% |
| 772 | L | 76 | SYNCHRONY 2 | FLEX28 | SONNET 2 | 1-year | 100% |
| 772 | R | 79 | SYNCHRONY 2 | FLEX28 | SONNET 2 | 3-months | 100% |
| 793 | L | 44 | SYNCHRONY | FLEX28 |  | pre-OP | 80% |
| 813 | R | 55 | SYNCHRONY | FLEX24 |  | pre-OP | 100% |
| 815 | R | 72 |  | STANDARD | SONNET 2 | 3-months | 100% |
| 861 | L | 59 | SYNCHRONY 2 | FLEX26 | SONNET 2 | 3-months | 100% |
| 861 | R | 59 | SYNCHRONY | FLEX26 |  | pre-OP | 100% |
| 874 | R | 78 | SYNCHRONY | FLEX28 | SONNET | pre-OP | 90% |
| 889 | L | 55 | SYNCHRONY 2 | FLEX26 | SONNET 2 | 3-months | 90% |
| 929 | L | 70 | SYNCHRONY 2 | FLEX24 | SONNET 2 | 3-months | 10% |
| 929 | L | 70 | SYNCHRONY 2 | FLEX24 | SONNET 2 | 6-months | 100% |
| 937 | R | 42 | CONCERTO | FLEX28 | SONNET 2 | pre-OP | 70% |
| 937 | R | 42 | CONCERTO | FLEX28 | SONNET 2 | 3-months | 90% |
| 954 | R | 25 | SYNCHRONY 2 | FLEX28 | SONNET 2 | 3-months | 90% |
| 973 | L | 80 | SYNCHRONY | FLEX28 |  | pre-OP | 60% |
| 980 | L | 88 | SYNCHRONY | FLEX28 |  | pre-OP | 50% |
| 985 | R | 71 | SYNCHRONY 2 | FLEX26 |  | pre-OP | 80% |
| 985 | R | 71 | SYNCHRONY 2 | FLEX26 | SONNET 2 | 3-months | 100% |
| 985 | R | 71 | SYNCHRONY 2 | FLEX26 | SONNET 2 | 6-months | 100% |
| 1010 | L | 66 | SYNCHRONY 2 | FLEX28 | SONNET 2 | 3-months | 60% |
| 1010 | L | 66 | SYNCHRONY 2 | FLEX28 | SONNET 2 | 6-months | 90% |
| 1023 | R | 73 | SYNCHRONY 2 | FLEX28 | SONNET 2 | 3-months | 100% |
| 1023 | R | 73 | SYNCHRONY 2 | FLEX28 | SONNET 2 | 6-months | 80% |
| 1043 | L | 62 | SYNCHRONY 2 | FLEX28 | SONNET 2 | pre-OP | 90% |
| 1043 | L | 62 | SYNCHRONY 2 | FLEX28 | SONNET 2 | 6-months | 100% |
| 1054 | L | 14 | SYNCHRONY 2 | FLEX28 | SONNET 2 | 3-months | 90% |
| 1085 | L | 71 | SYNCHRONY | FLEX28 | SONNET | pre-OP | 30% |
| 1094 | L | 64 | SYNCHRONY | FLEX28 |  | pre-OP | 60% |
| 1097 | R | 62 | SYNCHRONY 2 | FLEX26 |  | pre-OP | 20% |
| 1097 | R | 62 | SYNCHRONY 2 | FLEX26 | SONNET 2 | 3-months | 100% |
| 1097 | R | 62 | SYNCHRONY 2 | FLEX26 | SONNET 2 | 1-year | 100% |
| 1115 | R | 64 | SYNCHRONY 2 | FLEX28 | SONNET 2 | 3-months | 100% |
| 1115 | R | 64 | SYNCHRONY 2 | FLEX28 | SONNET 2 | 6-months | 100% |
| 1115 | R | 64 | SYNCHRONY 2 | FLEX28 | SONNET 2 | 1-year | 90% |
| 1117 | R | 86 | SYNCHRONY 2 | FLEX28 | SONNET 2 EAS | 3-months | 70% |
| 1135 | R | 59 | SYNCHRONY 2 | FLEXSOFT | SONNET 2 | 6-months | 90% |
| 1162 | R | 56 |  | FLEXSOFT | SONNET 2 | 3-months | 60% |
| 1172 | R | 61 |  | FLEXSOFT | SONNET 2 | 3-months | 70% |
| 1173 | L | 80 | SYNCHRONY 2 | FLEX28 | SONNET 2 | 3-months | 100% |
| 1173 | L | 80 | SYNCHRONY 2 | FLEX28 | SONNET 2 | 6-months | 100% |
| 1181 | R | 42 | SYNCHRONY | FLEX24 |  | pre-OP | 89% |
| 1202 | R | 51 | SYNCHRONY 2 | STANDARD | SONNET 2 | 3-months | 89% |
| 1202 | R | 51 | SYNCHRONY 2 | STANDARD | SONNET 2 | 6-months | 90% |
| 1252 | L | 31 | SYNCHRONY 2 | FLEX28 | SONNET 2 | 3-months | 90% |
| 1252 | R | 30 | SYNCHRONY | FLEX28 |  | pre-OP | 50% |
| 1301 | R | 64 |  | FLEX28 | SONNET 2 EAS | 3-months | 100% |
| 1307 | R | 85 | SYNCHRONY 2 | FLEX28 |  | pre-OP | 0% |
| 1307 | R | 85 | SYNCHRONY 2 | FLEX28 | SONNET 2 EAS | 3-months | 100% |
| 1307 | R | 85 | SYNCHRONY 2 | FLEX28 | SONNET 2 EAS | 6-months | 100% |
| 1329 | L | 80 |  | FLEXSOFT | SONNET 2 EAS | 3-months | 70% |
| 1344 | R | 30 | SYNCHRONY 2 | STANDARD | SONNET 2 | 3-months | 90% |
| 1399 | R | 44 |  | FLEXSOFT | SONNET 2 | 3-months | 70% |
| 1401 | R | 53 | SYNCHRONY | FLEX28 |  | pre-OP | 100% |
| 1425 | L | 79 | SYNCHRONY 2 | STANDARD | SONNET 2 | 3-months | 40% |
| 1436 | R | 59 | CONCERTO | FLEX24 | SONNET | pre-OP | 90% |
| 1438 | L | 29 | SYNCHRONY 2 | FLEX28 | SONNET 2 | 3-months | 70% |
| 1438 | L | 29 | SYNCHRONY 2 | FLEX28 | SONNET 2 | 6-months | 100% |
| 1454 | R | 88 | SYNCHRONY | FLEX28 | SONNET | pre-OP | 80% |
| 1461 | L | 18 | SYNCHRONY | FLEX28 |  | pre-OP | 0% |
| 1485 | R | 59 | SYNCHRONY 2 | FLEX28 |  | pre-OP | 100% |
| 1485 | R | 59 | SYNCHRONY 2 | FLEX28 | SONNET 2 | 3-months | 100% |
| 1488 | L | 37 | SYNCHRONY 2 | FLEX28 | SONNET 2 | 3-months | 80% |
| 1488 | L | 37 | SYNCHRONY 2 | FLEX28 | SONNET 2 | 6-months | 90% |
| 1570 | L | 58 | SYNCHRONY 2 | FLEXSOFT |  | pre-OP | 50% |
| 1570 | L | 58 | SYNCHRONY 2 | FLEXSOFT | SONNET 2 | 3-months | 90% |
| 1622 | R | 78 | SYNCHRONY 2 | FLEX28 | SONNET 2 | 6-months | 100% |
| 1657 | L | 52 | SYNCHRONY 2 | FLEX24 |  | pre-OP | 100% |
| 1657 | L | 52 | SYNCHRONY 2 | FLEX24 | SONNET 2 | 3-months | 100% |
| 1669 | L | 63 | SYNCHRONY 2 | FLEX28 | SONNET 2 | 3-months | 90% |
| 1669 | L | 63 | SYNCHRONY 2 | FLEX28 | SONNET 2 | 6-months | 80% |
| 1669 | L | 63 | SYNCHRONY 2 | FLEX28 | SONNET 2 | 1-year | 90% |
| 1680 | L | 76 | SYNCHRONY | FLEX24 |  | pre-OP | 90% |
